# Microwave-Assisted Rapid Preparation of Nano-ZnO/Ag Composite Functionalized Polyester Nonwoven Membrane for Improving Its UV Shielding and Antibacterial Properties

**DOI:** 10.3390/ma11081412

**Published:** 2018-08-11

**Authors:** Dongfeng Shao, Qufu Wei

**Affiliations:** 1 Changzhou Vocational Institute of Textile and Garment, Changzhou 213164, China; syx3313@163.com; 2 Key Laboratory of Eco-Textiles, Ministry of Education, Jiangnan University, Wuxi 214122, China

**Keywords:** polyester, ZnO, Ag, microwave assistant

## Abstract

The cost and efficiency of preparing ZnO/Ag composite functional polyester membrane affect their application, for which a rapid microwave-assisted method was studied for coating ZnO/Ag composite nanoparticles on polyester nonwoven. The surface morphology, crystalline structure, and surface chemistry of the uncoated and coated polyester nonwoven was investigated by X-ray diffractometer (XRD), scanning electron microscopy (SEM), Fourier transform infrared spectroscopy (FT-IR), energy-dispersive spectroscopy (EDS), X-ray photoelectron spectroscopy (XPS), and thermogravimetric (TG), respectively. Washing stability, ultraviolet properties, and antibacterial properties of before and after treatment polyester nonwoven were also investigated. The results indicated that Ag/ZnO composite nanoparticles were successfully deposited on polyester nonwoven surface. The amount of silver nitrate added in reaction has an important effect on the morphology and structure of Ag/ZnO composite on the surface of polyester fiber. The washing experiment results show that the ZnO/Ag composite functional polyester nonwoven fabric prepared by this method exhibits good washing durability after 90 min of washing. The results of UV transmission analysis showed that polyester nonwoven has an obvious increase in ultraviolet resistant properties after Ag/ZnO composite coating. When 0.2 g of silver nitrate was added into 100 mL of the reaction solution, the mean ultraviolet protection factor (UPF) of the treated polyester nonwoven reached a maximum of 219.8. The antibacterial results showed that the coated nonwoven against *Escherichia coli* and *Staphylococcus aureus* was about 94.5% and 96.6%, respectively, showing very good antibacterial properties.

## 1. Introduction

Polyester nonwoven are widely used in automotive interior decoration, garment linings, medical materials, and filter materials because of their high flexibility, good thermal stability, corrosion resistance, and strong mechanical properties [1]. However, some shortcomings, such as poor hygroscopicity, easily generates static charge, and lack of antibacterial and UV shielding properties, affect its application effect.

In recent years, nano ZnO/Ag composite functional polyester nonwoven has been widely concerned because of its excellent optical and electrical properties. Nano ZnO/Ag composite not only endow UV shielding, antistatic, anti-bacterial, and photocatalytic properties to polyester fabric [2,3], but also extend the application of polyester fabric in flexible transparent electrode [4,5,6] and flexible nanogenerators [7]. Magnetron sputtering [8,9] and hydrothermal methods [10] have been successfully used to prepare nano ZnO/Ag composite functional polyester fabric. However, among these methods, magnetron sputtering requires two sputtering targets and many operational steps. Hydrothermal method needs to deposit ZnO seed or indium tin oxides (ITO) layer on the surface of polyester, and then nano ZnO/Ag composite can grow on ZnO seed or ITO layer under hydrothermal conditions, which take a long time. Therefore, it is necessary to study a simple and rapid method for preparing nano ZnO/Ag composite functional polyester nonwoven to meet the needs of industry. 

Different technologies have been used to study the rapid preparation of nano ZnO or nano silver functional textiles. Microwave-assisted has received wide attention because of some unique advantages. For example, there is no direct contact between the reaction and the energy source during microwave heating, which acts directly on the molecule, and the heating rate is fast, the reaction is uniform, and so on [11]. It is successfully used to prepare ZnO functional cotton fabric [12,13], silver nanoparticles coated bamboo pulp fabric [14], and silver nanoparticles deposited viscose fibers [15] in a short time. In addition, a one-step preparation method of nano ZnO or nano Ag functional polyester has been reported. Hajar Poortavasoly, et al., reported that polyester fabric was surface modified with triethanolamine and simultaneously surface deposited ZnO at 130 °C about 1 h [16]. Also, nano Ag was deposited on the polyester fabric surface with the same method under the same conditions [17]. These studies provide a good idea for us to prepare nano ZnO/Ag composite functional polyester nonwoven quickly. Polyester nonwoven surface can be treated by triethanolamine and simultaneously nano ZnO/Ag composite be rapidly deposited on polyester nonwoven surface with microwave assistant.

In this study, this method is used for the rapid preparation of nano ZnO/Ag composite functional polyester nonwoven. The surface morphology, and physical and chemical structure of polyester nonwoven before and after treatment were investigated. Washing stability, ultraviolet properties, and antibacterial properties of polyester nonwoven after treatment were also studied.

## 2. Materials and Methods

### 2.1. Materials

Meltblown polyester nonwoven (52 g/m^2^) was provided by Jiangsu Chuangyuan Interlinings Co., Ltd., Jiangsu, China. Samples were washed with ethanol and distilled water before use. Zinc nitrate hexahydrate, silver nitrate, and triethanolamine were purchased from Shanghai Chemical Reagent Co. shanghai, China. All the reagents were used as received. *Staphylococcus aureus* (ATCC 6538) and *Escherichia coli* (ATCC 8099) was from the Analysis and Testing Center of College of Textile and Clothing Engineering of Soochow University (Soochow, China). 

### 2.2. Preparation of ZnO–Ag Composite Coated Polyester Nonwoven

**Figure 1:**
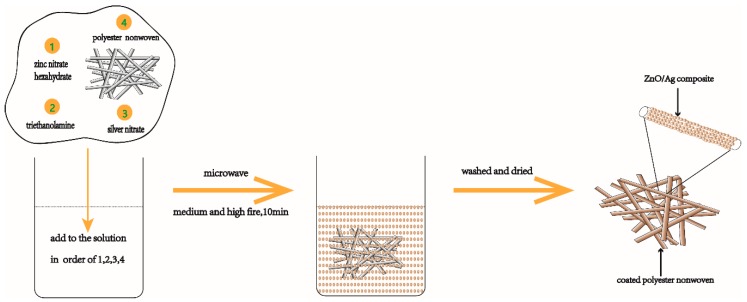
Schematic of rapid ZnO/Ag composite functionalization of polyester nonwoven by microwave-assisted.

ZnO–Ag composite coated polyester nonwoven was prepared as is illustrated in Figure 1. First, 2.97 g zinc nitrate hexahydrate was first dissolved in 80 mL distilled water under stirring. Then, 20 mL triethanolamine was slowly added, and stirred at room temperature to obtain a clear solution. Next, 0.1 g–0.3 g silver nitrate was dissolved in the above solution under stirring. Samples of various treatments are shown in Table 1. Further, a 6 cm × 6 cm polyester nonwoven sample was immersed into the mixture solution, and then placed into the microwave oven (P80D23N1L, Galanz, Shunde, China) with a frequency of 2450 MHz and a power of 800 W. The reaction was performed for 10 min in the microwave oven under medium and high fire. During the microwave treatment, checked every two minutes to make sure that the sample was immersed in the solution and kept the sample flat and wrinkle free. Finally, the treated sample was taken out of the solution, washed with distilled water, and then dried at 80 °C.

### 2.3. Characterizations

Scanning electron microscopy (SEM, S-4800, Hitachi, Tokyo, Japan) was used to examine the surface morphology of the polyester nonwoven and ZnO/Ag composite coated polyester nonwoven. The magnification of the SEM is 2000 and 20,000, respectively. 

X-ray diffractometer (XRD, D/max2500PC, Rigaku, Tokyo, Japan) was used to investigate the phase and crystalline state of before and after treatment polyester nonwoven. The XRD patterns were recorded using a Cu Karadiation (I = 1.54056 Å). 

Surface chemical of before and after treatment polyester nonwoven was examined by Fourier transform infrared spectroscopy (FT-IR, IS50, ThermoFisher, MA, USA), energy-dispersive spectroscopy (EDS, quantax400, Bruker, Berlin, Germany), and X-ray photoelectron spectroscopy (XPS, Escalab 250Xi, ThermoFisher, MA, USA). The FT-IR absorption spectrum was recorded in the range of 4000–400 cm^−1^ with 32 scans at a resolution of 4 cm^−1^. Energy-dispersive spectroscopy was used to characterize the elemental distribution of after treatment polyester nonwoven. XPS were recorded using amonochromated Al Kα X-ray source (1486.7 eV). The size of the X-ray beam was 200 μm.

Thermogravimetric (TG, Q500, TA Instruments, DE, USA) is used to detect the thermal stability of the treated samples. Thermo gravimetric measurements are performed from 50 to 595 °C using a linear heating rate of 10 °C/min in a nitrogen atmosphere.

### 2.4. Washing Stability

Washing stability of ZnO/Ag composite coated polyester nonwoven was assessed by washing experiment using a domestic washing machine. The samples were dried after washing for 30, 60, and 90 min, then weighed, after which the washing stability was evaluated. A typical 30 min washing procedure was as follows: 10 g ZnO/Ag composite coated polyester nonwoven washed with 5 g of standard detergent in 4 L of water for 30 min (30 °C). It was dehydrated and then washed with 4 L of water for 2 min under room temperature. Finally, the sample was dried at 60 °C. The washing stability was evaluated by the reduction percentage of weight between the washed sample and control sample.

### 2.5. Ultraviolet Resistant Properties

Ultraviolet transmittance analyzer (UV-1000F, Lapsphere, NH, USA) was used to test ultraviolet resistant properties of polyester nonwoven before and after coating according to GB/T 18830-2009 [18]. The ultraviolet transmittance was recorded in the range of 290–400 nm. The results of ultraviolet resistant property were evaluated by solar UV-A spectral transmittance (T(UVA)), solar UV-B spectral transmittance (T(UVB)), and ultraviolet protection factor (UPF). Each sample was tested four times at different areas, and the average values were obtained. 

### 2.6. Antibacterial Properties

The antibacterial properties of before and after treated polyester nonvowen were evaluated using the shake flask method against *Staphylococcus aureus* and *Escherichia coli*. Each fresh bacterial strain was cultured under the conditions of nutrient agar (37 °C, 18 h). Then, inoculate fresh bacteria in a nutrient solution at 37 °C for 18 h, and diluted to a suspension (1 × 10^4^ ~ 9 × 10^4^ CFU/mL) with phosphate buffer saline. CFU is colony forming units. The treated polyester samples and control samples were each cut into a size of 0.5 cm × 0.5 cm and sterilized in an autoclave at 121 °C for 15 min. Next, 70 mL phosphate buffered saline, 0.75 g sample, and 5 mL the diluted bacteria suspension were sequentially added to a erlenmeyer flask (250 mL). It was then shaken at 150 rpm for 18 h at room temperature. Next, 1 mL bacteria solution collected from each erlenmeyer flask was diluted 10 times, 100 times, and 1000 times, respectively. Finally, bacterial suspension of different dilutions was inoculated on agar medium for 24 h at 37 °C, and colonies were counted. The antibacterial properties were evaluated by the reduction percentage of bacteria colonies between the treated sample and control samples. 

## 3. Results and Discussion

### 3.1. Crystalline Structure Analysis

**Figure 2:**
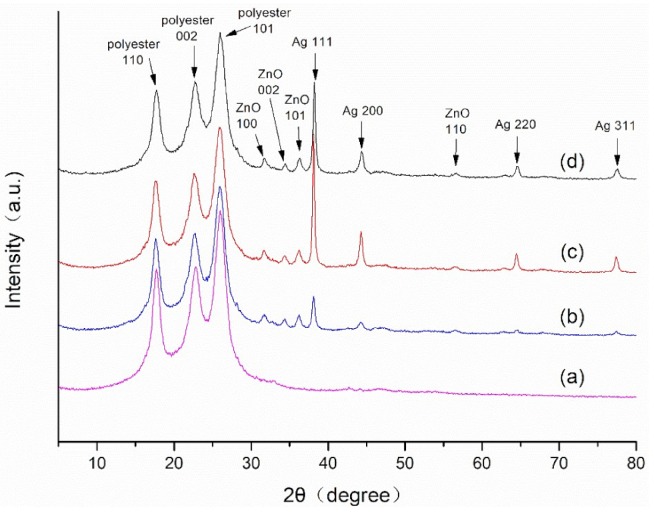
X-ray diffractometer (XRD) patterns of (**a**) polyester nonwoven; (**b**) ZnO/Ag composite coated polyester nonwoven (3#); (**c**) ZnO/Ag composite coated polyester nonwoven (4#); and (**d**) ZnO/Ag composite coated polyester nonwoven (5#).

Figure 2a shows the X-ray diffraction (XRD) patterns of polyester nonwoven. As is shown in Figure 2a, the reflection peaks at 2θ = 17.4, 22.7 and 25.4 correspond to (100), (002), and (101) planes of polyester nonwoven, respectively. Figure 2b–d are all XRD patterns of ZnO/Ag composite coated polyester nonwoven. Their difference is that the amount of silver nitrate added in the preparation process is 0.1 g, 0.2 g, and 0.3 g, respectively. From Figure 2b, it can be seen that a series of peaks at 2θ = 31.7°, 34.4°, 36.2°, 38.1°, 44.3°, 56.6°, 64.4°, and 77.5° are detected in the XRD curve, besides the reflection peaks of polyester nonwoven. The peak at 2θ = 31.7°, 34.4°, 36.2°, and 56.6° contribute to (100), (002), (101), and (110) planes of the hexagonal wurtzite ZnO, respectively (JCPDS 36-1451) [19]. The peak appearing at 2θ = 38.1° and 44.3°, 64.4°, and 77.5° correspond to the (111), (220), (200), and (311) crystal plane of silver, respectively (JCPDS 04-0783) [19]. These results indicate that the ZnO/Ag composite is successfully deposited on the surface of polyester nonwoven under this conditions. Also, it can be seen that the reflection peak intensity of the (100), (002), and (101) crystal planes of polyester nonwoven decreases slightly, which is attributed to the decrease in crystallinity of polyester nonwoven caused by the reaction of triethanolamine with polyester. Compared with Figure 2b, it can be observed that there is the increase of the reflection peak intensity of the (111), (220), (200), and (311) crystal plane of silver, as is shown in Figure 2c,d. This is a result of the increase in silver nitrate content in the reaction, and more silver is reduced by triethanolamine, causing crystal growth of silver on the nonwoven surface. In addition, it can be seen that the crystallinity of silver in the ZnO/Ag composite deposited on the nonwoven surface in Figure 2c is better than that of the silver in Figure 2d. This implies that the amount of silver nitrate affects the structure of ZnO/Ag composite on the nonwoven surface.

### 3.2. Surface Morphology Analysis

**Figure 3:**
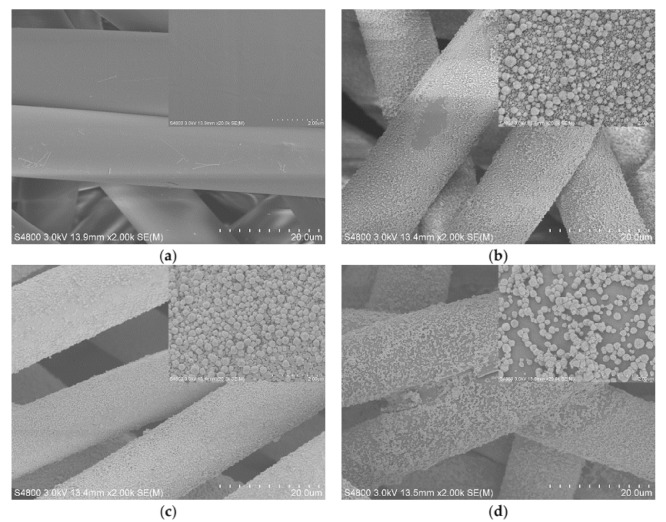
Scanning electron microscopy (SEM) images of (**a**) polyester nonwoven; (**b**) ZnO/Ag composite coated polyester nonwoven (3#); (**c**) ZnO/Ag composite coated polyester nonwoven (4#); and (**d**) ZnO/Ag composite coated polyester nonwoven (5#).

The SEM images of polyester nonwoven are shown in Figure 3a. It can be seen that the polyester fiber surface appears to be relatively smooth, and a small number of elongated rods are distributed on the fiber surface. Compared with untreated polyester fiber, it can be observed that the surface of the polyester fiber showed significant changes after ZnO/Ag composite coating, as is illustrated in Figure 3b–d. From Figure 3b, it can be seen that most of the surface of the fiber is evenly wrapped by particles. These particles are divided into two layers. The bottom particles are irregular granules with the size range of 50–260 nm, but the upper part is spherical particles with the size of 110–380 nm, and the average diameter is about 320 nm. The irregular particles are because the reaction of triethanolamine with the polyester will be grafted onto the surface of the fiber [17]. Similar to the principle that C_2_H_2_O_4_ controls the formation of irregular block ZnO [20], several hydroxyl groups grafted on the fiber surface adsorb Zn^2+^ form templates and control irregular ZnO granules generation on the surface of the fiber. When the fiber surface triethanolamine is covered with particles and the silver ions are reduced to Ag on the surface of the particles, some ZnO seeds are then deposited on the surface of Ag [21]. With the increase of the reaction time, ZnO grows on the seeds to form the upper spherical ZnO/Ag composite structure. However, under the condition of 0.2 g silver nitrate being added, the particles coated on the surface of the fiber are more uniform, as is illustrated in Figure 3c. These particles also have two layers, but the size of spherical particles in the upper layer ranges from 170 nm to 330 nm, and the average particle size is about 260 nm. When 0.3 g of silver nitrate was added, the surface of the fiber had only spherical composite particles with sizes ranging from 150 to 440 nm, and the average size is about 270 nm, as is shown in Figure 3d. This is because the silver nitrate content in the solution increases, and more silver ions are reduced to silver on the fiber surface, which hinders the effect of triethanolamine on the growth morphology of ZnO/Ag composite on the fiber surface. Combined with previous XRD analysis and SEM image, it was further proved that the amount of silver nitrate added in the coating reaction had a significant effect on the structure of the ZnO/Ag composite particles deposited on the nonwoven surface.

### 3.3. Surface Chemistry Analysis

**Figure 4:**
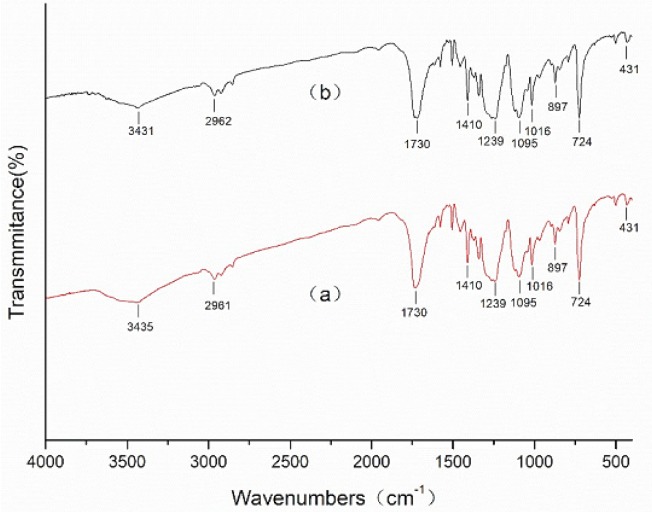
Fourier transform infrared spectroscopy (FTIR) spectra of (**a**) polyester nonwoven; (**b**) triethanolamine treated polyester nowoven (1#).

**Figure 5:**
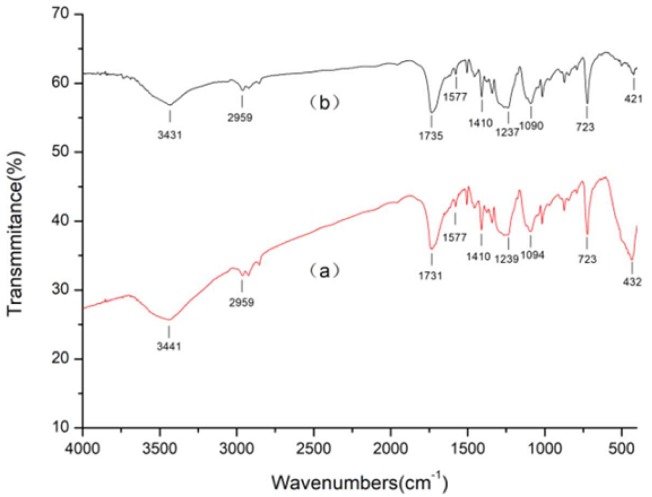
FTIR spectra of (**a**) ZnO coated polyester nonwoven (2#); (**b**) ZnO/Ag composite coated polyester nowoven (3#).

In order to explore the mechanism of ZnO/Ag composite functional polyester, the infrared spectra of polyester, triethanolamine treated polyester, ZnO coated polyester, and ZnO/Ag composite coated polyester were analyzed, respectively. Figure 4 shows the FT-IR spectra of polyester nonwoven and triethanolamine treated polyester nonwoven. As is shown in Figure 4a, the band at 3439 cm^−1^ is ascribed to –OH group, while those at 2961 cm^−1^ and 1454 cm^−1^ are assigned to the C–H stretching vibration [22]. The band at 1730 cm^−1^ is attributed to the stretching vibration of the C=O bond. The band at 1577 cm^−1^ is represented to the C–H bond stretching vibration of the phenyl ring. The band at 1410 cm^−1^ is attributed to the C–C phenyl ring stretching. The band at 1239 cm^−1^ and 1095 cm^−1^ is due to C–O stretching [23]. From Figure 4b, it can be seen that the band of triethanolamine treated polyester nonwoven changed at 3431 cm^−1^, 2921 cm^−1^, and 1735 cm^−1^ compared with that of polyester nonwoven. This is due to the treatment of the polyester by triethanolamine, which causes some of the triethanolamine molecules to be grafted onto the surface of the polyester [17], causing subtle changes in the bonds such as C=O, C–H, and O–H. Figure 5a shows the FT-IR spectra of ZnO coated polyester. Compared with the spectra of polyester nonwoven and triethanolamine treated polyester nonwoven, there are obvious shifts in the band at 3441 cm^−1^, 2959 cm^−1^, 1730 cm^−1^, 1239 cm^−1^, 1094 cm^−1^, and 723 cm^−1^, and the new band is found at 432 cm^−1^, as shown in Figure 5a. The new band at 432 cm^−1^ was attributed to the band of Zn–O vibrations [24]. Other infrared absorption peak shifts are due to polyester ester groups reacting with hydroxyl groups of triethanolamine. However, after ZnO/Ag composite coated polyester nonwoven, the band of C=O stretching vibration bond at 1735 cm^−1^, C–O stretching bond at 1237 cm^−1^ and 1090 cm^−1^ are shifted, as is shown in Figure 5b. This is because the hydroxyl of triethanolamine reacts with silver nitrate to form the aldehyde group (C=O), and the aldehyde group may continue to react with silver nitrate to form a carboxyl group (O=C–O). Also, the band of Zn–O vibrations at 432 cm^−1^ obviously weakened and offset to 421 cm^−1^, which may be because silver doped ZnO affects the Zn–O vibrations.

**Figure 6:**
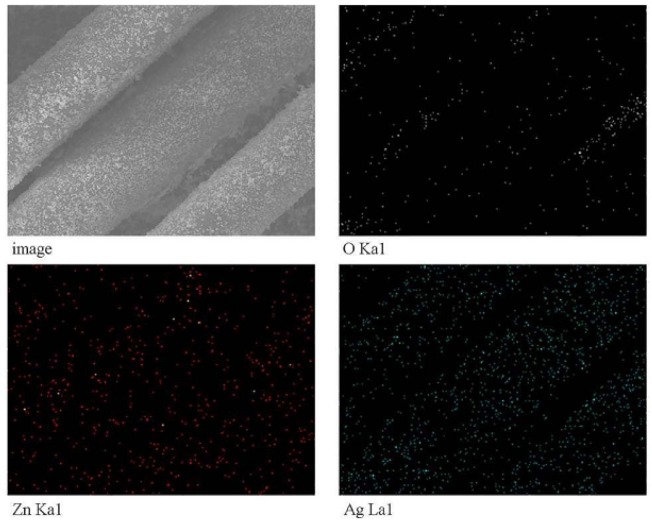
X-ray mapping images of ZnO/Ag composite coated polyester nonwoven (4#).

X-ray mapping images of ZnO/Ag composite coated polyester nonwoven (4# sample) are presented in Figure 6. It can be seen that the zinc element is evenly distributed on the fiber surface and the fiber gap, while silver elements are mainly distributed on the surface of fibers. This indicates that Ag might be encapsulated by ZnO on the nonwoven surface [25].

**Figure 7:**
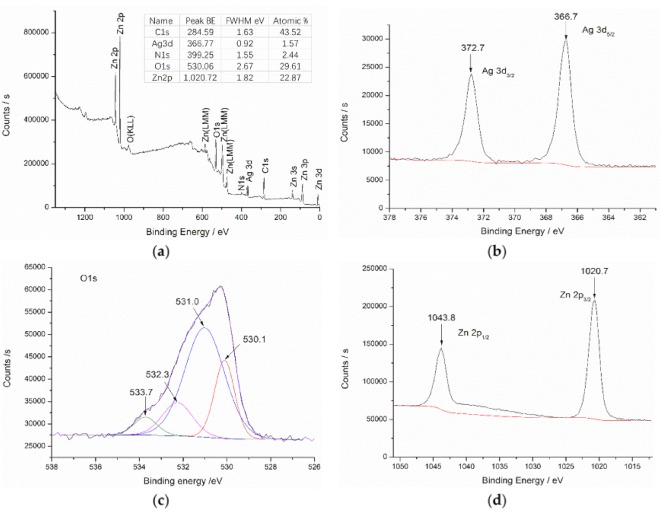
X-ray photoelectron spectroscopy of ZnO/Ag composite coated polyester nonwoven (4#) (**a**) full spectrum; (**b**) Ag3d peak; (**c**) O1s peak; and (**d**) Zn2p peak.

XPS analysis of the content and valence of each element in the ZnO/Ag composite on the surface of nonwoven is crucial to the study of the properties of the materials, just like the surface of nitrogen-coated silver nanoparticles was analyzed by XPS and its antibacterial activity was explored [26]. Figure 7 presents X-ray photoelectron spectroscopy of ZnO/Ag composite coated polyester nonwoven prepared with 0.2 g silver nitrate. From Figure 7a, it can be seen that there are five characteristic peaks of C, Ag, N, O, and Zn from the full spectrum. Among them, the atomic content percentages of C1s, Ag3d, N1s, O1s, and Zn2p were 43.52%, 1.57%, 2.44%, 29.61%, and 22.87%, respectively. The high-resolution XPS spectra of Ag3d and Zn2p in the Ag/ZnO composite coated polyester nonwoven are shown in Figure 7b,c, respectively. It can be seen that two peaks centered at 366.7 eV and 372.7 eV as is shown in Figure 7b, which can be attributed to Ag 3d_5/2_ and Ag 3d_3/2_, respectively. The difference of binding energy between 3 Ag 3d_5/2_ and Ag 3d_3/2_ is 6.0 eV, which further confirms the formation of elemental silver on the surface of polyester nonwoven [27]. In addition, the peak positions of Ag 3d_5/2_ and Ag 3d_3/2_ are lower than the standard binding energy of Ag 3d for bulk Ag, which is mainly attributed to the migration of electrons between Ag and ZnO crystals [21]. In Figure 7c, the O1s profile can be fitted into four peaks. The peak at 530.0 eV and 532.3 eV are attributed to the lattice oxygen and surface hydroxyl oxygen of ZnO, respectively [27]. The peak at 533.7 eV is associate with C–O bonds [28]. The peak at 531.0 eV is relate to C=O bonds. It is lower than the C=O binding energy of the polyester because the charge transfers from the Ag atom to the O=C functional group of the ester group leads to a decrease in binding energy [29]. From Figure 7d, high-resolution XPS spectra of Zn 2p3/2 and Zn 2p1/2 was observed at 1021.6 eV and 1044.8 eV. This is similar to the energy band of pure ZnO nanorods [21], which was also confirmed ZnO was deposited on the surface of the polyester. 

### 3.4. Thermogravimetric Analyses

**Figure 8:**
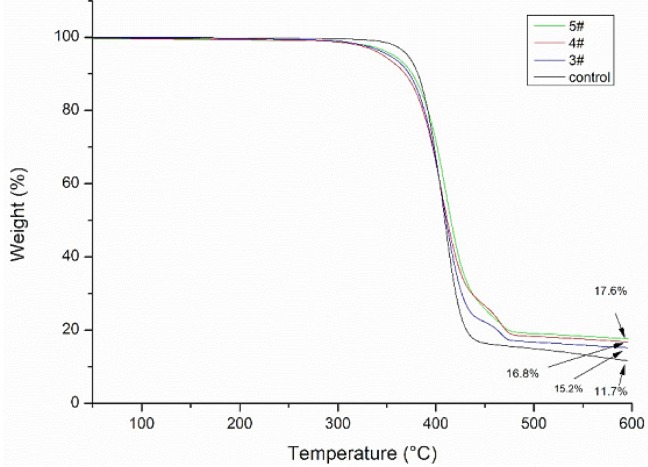
Thermogravimetric (TG) curves of control and ZnO/Ag composite coated polyester nonwoven.

Figure 8 presents the TG curves for the control sample, ZnO/Ag composite coated polyester nonwoven (3# sample, 4# sample, and 5# sample). It is clearly observed that the TG curves of the control sample includes one weight loss step. Although that of ZnO/Ag composite coated polyester nonwoven includes 50–494 °C weight loss stages and 494–595 °C weight loss stages, which can be attributed to the reaction between ZnO/Ag composite with polyester thermal decomposition products. The coated polyester nonwoven show an initial loss greater than those of control sample because the triethanolamine treatment causes the macromolecular chain breakage of polyester, which is easily decomposed by heat. In the latter stage, the weight loss of the sample is less than the original. The thermal weight loss of the control sample, 3# sample, 4# sample, and 5# sample at 595 °C reached 88.3%, 84.8%, 83.2%, and 82.4%, respectively. This is attributed to the fact that the ZnO/Ag composite deposited on the surface of the polyester nonwoven is not easily decomposed, resulting in a reduction in weight loss.

### 3.5. Washing Stability Properties

Weight changes of the ZnO/Ag coated polyester nonwoven washed over different time is shown in Table 2. Comparing the samples of 3#, 4#, and 5#, it can be seen that when 0.2 g silver nitrate was added to the reaction, the weight increase of the treated sample was the highest, reaching 0.0186 g.

This indicates that more ZnO/Ag composite are deposited on the surface of the fabric under this condition. After 30 min washing, it was observed that the weight loss of the 4# sample was the largest and the 5# sample was the smallest. This is because the 4# sample forms two layers of different shape particles on the surface of the fiber, the force between the particles is weak, and it falls off easily during washing. However, the surface particles of 5# sample are sparse, and the particles act directly on the fibers, so the weight loss is minimal. Similar phenomena were also observed on weight loss of the ZnO/Ag composite coated polyester nonwoven after 60 min and 90 min of washing. However, even if it was washed for 90 min, the Zn/Ag composite deposited on the surface of the nonwoven of 3#, 4#, and 5# sample left 0.01 g, 0.0094 g, and 0.0129 g, respectively, which was more than half of the surface deposition weight of the polyester nonwoven. This shows that the ZnO/Ag composite functional polyester nonwoven prepared by this method has very good washing durability.

### 3.6. Ultraviolet Resistant Properties

Table 3 shows the ultraviolet resistant properties of untreated polyester nonwoven and treated polyester nonwoven. From Table 3, it can be observed that the mean UPF of untreated polyester nonwoven is only 14.6. Although the ultraviolet resistant properties of polyester nonwoven increased sharply after ZnO/Ag composite coating because the UPF of the lowest coated nonwoven reached 166.8, which is a result of the strong UV light absorption of ZnO/Ag composite for the coated polyester nonwoven. Comparing 3#, 4#, and 5# samples, it can be seen that under the condition of 0.2 g silver nitrate, the average UPF of the ZnO/Ag composite coated nonwoven is higher than that of the condition of 0.1 g or 0.3 g silver nitrate. According to the previous thermogravimetric analysis and weighing before the water washing test, this is mainly due to the greater amount of Zn/Ag composite coated on the nonwoven surface.

### 3.7. Antibacterial Properties

**Figure 9:**
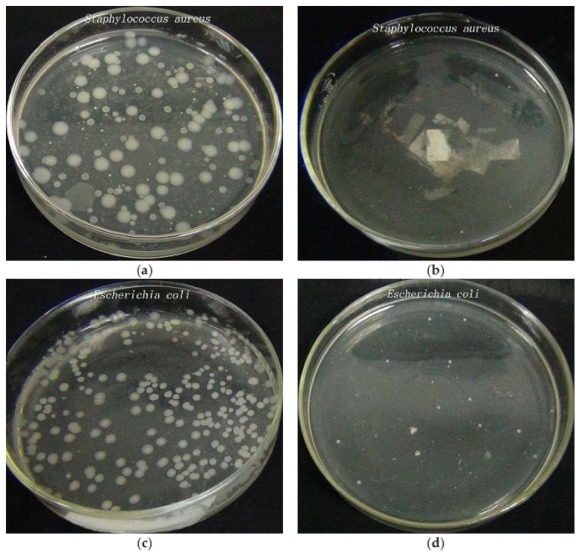
Shake flask antimicrobial test results of (**a**) control sample against *S. aureus*; (**b**) ZnO/Ag coated polyester nonwoven (4#) against *S. aureus*; and (**c**) control sample against *E. coli*; (**d**) ZnO/Ag coated polyester nonwoven (4#) against *E. coli*.

Different methods can be used to test the antimicrobial properties of nanocomposite functional materials, such as the agar diffusion plate method and the shake flask method [30]. The agar diffusion plate method checks whether the product has antibacterial properties, while the shake flask method gives more accurate antibacterial results. Therefore, the shake flask method was used to examine the antimicrobial properties of zinc oxide/silver composite coated polyester nonwovens in this study. Figure 9 shows the antibacterial test results of control sample and ZnO/Ag composite coated polyester nonwoven after dilution of the bacterial solution 1000 times. From Figure 9a,b, it can be seen that the the control sample inoculated with *S. aureus* bacteria showed bacterial count about 146 CFU, while that of treated polyester nonwoven was about 5 CFU. The reduction percentage of *Staphylococcus aureus* between ZnO/Ag composite coated polyester nonwoven and control sample is about 96.6%. Similarly, the count of *E. coli* colonies cultured with control sample is about 235 CFU, and that of *E. coli* colonies with coated fabric is about 13 CFU, as shown in Figure 9c,d. This indicates that the coated fabric has an antibacterial rate of 94.5% for *E. coli*. These results indicate that ZnO/Ag composite functional polyester nonwoven has good antibacterial properties, which are attributed to the inhibition of bacterial growth by the release of Ag^+^ and Zn^2+^ by the ZnO/Ag composite deposited on the polyester nonwoven surface, as well as by the formation of reactive oxygen species on ZnO surface [31,32].

## 4. Conclusions

The Ag/ZnO nanocomposite was successfully deposited on the polyester nonwoven with microwave assistance for 10 min. The experimental results showed that the amount of silver nitrate used in the reaction has an important influence on the morphology and structure of the Ag/ZnO composite deposited on the nonwoven surface. The results of TG indicated that the thermal stability of the treated nonwoven was better than that of the control sample because of the deposition of the ZnO/Ag composite. The results of the water washing experiment showed that the residual ZnO/Ag composite on the nonwoven after washing for 90 min exceeded half of that of the unwashed nonwoven. UV shielding performance test showed that the ZnO/Ag composite coated polyester nonwoven exhibited excellent UV shielding properties, especially when the amount of silver nitrate in the coating was 0.2 g. The results of antibacterial experiments showed that the ZnO/Ag composite functional polyester nonwoven has good antibacterial properties. All results indicate that this method has great potential in the production of Ag/ZnO composite functional textiles.

## Figures and Tables

**Table 1:** Samples prepared under different processing conditions.

Sample	H_2_O (mL)	Triethanolamine (mL)	Zn(NO_3_)_2_·6H_2_O (g)	AgNO_3_ (g)
Control	0	0	0	0
1#	80	20	0	0
2#	80	20	2.97	0
3#	80	20	2.97	0.1
4#	80	20	2.97	0.2
5#	80	20	2.97	0.3

**Table 2:** Weight change for the different washing times.

Sample	Treated Weight (g)	Treated Weight Gain (g)	30 min		60 min		90 min	
			Weight Loss (g)	Weight Loss (%)	Weight Loss (g)	Weight Loss (%)	Weight Loss (g)	Weight Loss (%)
3#	0.2134	0.0152	0.0015	0.70	0.0045	2.11	0.0052	2.44
4#	0.2213	0.0186	0.0052	2.35	0.0087	3.93	0.0092	4.16
5#	0.2103	0.0162	0.0003	0.14	0.0021	1.00	0.0033	1.57

**Table 3:** Ultraviolet resistant properties of sample.

Sample	Average T(UVA) (%)	Average T(UVB) (%)	Average UPF
Control	18.66	2.10	14.6
3#	7.46	0.12	166.8
4#	5.82	0.12	219.8
5#	6.22	0.14	197.4
